# A deadline constrained scheduling algorithm for cloud computing system based on the driver of dynamic essential path

**DOI:** 10.1371/journal.pone.0213234

**Published:** 2019-03-08

**Authors:** Xia Shao, Zhiqiang Xie, Yu Xin, Jing Yang

**Affiliations:** 1 College of Computer and Technology, Harbin University of Science and Technology, Harbin, Heilongjiang, China; 2 College of Computer and Technology, Harbin Engineering University, Harbin, Heilongjiang, China; University of Nevada, UNITED STATES

## Abstract

To solve the problem of the deadline-constrained task scheduling in the cloud computing system, this paper proposes a deadline-constrained scheduling algorithm for cloud computing based on the driver of dynamic essential path (Deadline-DDEP). According to the changes of the dynamic essential path of each task node in the scheduling process, the dynamic sub-deadline strategy is proposed. The strategy assigns different sub-deadline values to every task node to meet the constraint relations among task nodes and the user’s defined deadline. The strategy fully considers the dynamic sub-deadline affected by the dynamic essential path of task node in the scheduling process. The paper proposed the quality assessment of optimization cost strategy to solve the problem of selecting server for each task node. Based on the sub-deadline urgency and the relative execution cost in the scheduling process, the strategy selects the server that not only meets the sub-deadline but also obtains much lower execution cost. In this way, the proposed algorithm will make the task graph complete within its deadline, and minimize its total execution cost. Finally, we demonstrate the proposed algorithm via the simulation experiments using Matlab tools. The experimental results show that, the proposed algorithm produces remarkable performance improvement rate on the total execution cost that ranges between 10.3% and 30.8% under meeting the deadline constraint. In view of the experimental results, the proposed algorithm provides better-quality scheduling solution that is suitable for scientific application task execution in the cloud computing environment than IC-PCP, DCCP and CD-PCP.

## Introduction

Cloud computing has been increasingly developed on the basis of internet technologies, virtualization technologies, parallel processing technologies, distributed computing and grid computing. A payment method of “pay-per-use” is used by the cloud computing providers, which makes network service on-demand, scalable hardware and software. In recent years, cloud computing has become well developed. Because its user can purchase services through leasing way, and not buy a large number of hardware and software devices. In this situation, Cloud computing is put into use in different fields, such as electronics, economics and manufacturing. Reference [1–6] study and analyse cloud computing, grid computing, distributed computing and paralleling computing from multiple perspectives.

Cloud computing is a cloud service, which uses network and central controlling system to offer cloud service for different users. Amazon EC2, Tencent CVM, Google App Engine and Microsoft Azure are the existing prominent cloud servers. Virtualization is one of the key technologies of cloud computing, which is classified as full virtualization, OS-Layer virtualization, Hardware-layer virtualization, Para-Layer virtualization, Grid virtualization, Application-Layer virtualization, Resource virtualization, Storage virtualization, Cloud virtualization [7]. Cloud computing makes multiple virtual machines to reside a single physical computer system [8]. The cloud providers rend virtual machine to different users by paying-per-use-go [9]. Because the network resources and services owns the diversified, dynamic and flexible nature, the network service providers may offer the different service under meeting user’s defined QoS. Cloud services, which vary from person to person, are the advantage of cloud computing, and pose a new challenge for the development of scheduling algorithms in the cloud computing system [10, 11].

The cloud computing algorithms include resource management algorithms and workflow task scheduling algorithms. Resource management scheme is that how to rent the resources out to the cloud users on a pay-per-use basis to maximize the profit by achieving high resource utilization, Madni, et al investigate resource manage schemes and algorithms, and analysis and evaluates these schemes [12, 13]. The workflow task scheduling algorithm is a branch of cloud computing scheduling algorithms [14], which used to map task node to the suitable server, and of ordering the task nodes on each server to satisfy some performance criterion. Madni, et al present the comparison of heuristic algorithms for task scheduling [15]. The task-graph scheduling problem is an NP-hard optimization problem, and it difficult to achieve an optimal schedule result [16]. In recent years, some researchers had proposed many effective and feasible scheduling algorithms. The classical scheduling algorithm includes GBLCA(Global Leagure Championship Algorithm) [17] (Abdulhamid, S. M.et al.), dynamic clustering league championship algorithm (DCLCA) [18] (Abdulhamid, S. I. M, et al.), HEFT&CPOP [19] (Heterogeneous Earliest-Finish-Time)&(Critical-Path-on-a-Processor) (Topcuouglu H, et al), DLS(Dynamic Level Scheduling) [20] (Sih G C, et al), DSH(Duplication Scheduling Heuristic) [21] (Badawi, A A, et al.), FCBWTS(Workflow Task Scheduling Based on Fuzzy Clustering) [22] (Guo F Y, et al.), GA(Genetic Algorithm) [23] (Bonyadi M R, et al.), SA(Simulated Annealing) [24] (Dai M, et al.)etc. The QoS parameter of these algorithms is single, which is minimizing Makespan. In the cloud computing system, there are many important parameters, such as minimizing Makespan, minimizing the execution cost. The cloud servers own the different QoS parameters such as CPU type and memory size, and its price is different, for example, the server with faster CPU and more memory, its price is higher, in contrast, its price is lower. The scheduler must to consider a time-cost trade-off when they select server to schedule the workflow tasks, i.e., the multi-objective task graph scheduling in the cloud computing system. To address the multi-objective scheduling problem of task graph in the cloud computing system, many effective and feasible scheduling algorithms are proposed, which are classified heuristic and metaheuristic solutions [25]. The main concept of heuristic solution is that the feasible solution is given to solve the special condition problem, the time and space complexity of the solution is acceptable, but it difficult to achieve an optimal solution. The metaheuristic solution is a general heuristic solution, which solve the problem without the special condition, so the solution is widely applied. The classical metaheuristic solution contains PSO(Particle Swarm Optimizaiton)(Verma, A et al.) [26], ACO(Ant Colony Optimizaiton)(Daun W J et al.) [27], GA(Genetic Algorithm)(Verma, A et al.) [28], SA(Simulated Annealing)(Jian C f et al.) [29] and CSO(Cat Swarm Optimization) [30](Bilgaiyan S et al.). These algorithms own the higher time complexity and very higher time consuming, so they do not apply to the real cloud computing system sparingly.

Recently, many effective and feasible metaheuristic solutions are proposed. The main concept of metaheuristic solution that the reasonable scheduling order list of task nodes is acquired according to the property analysis of task graph, under the special constraints condition, such as deadline, budget etc., and map task node to the corresponding server. The classical heuristic solution includes IC-PCP&IC-PCPD2(Abrishami S et al.) [31] (IaaS Cloud Partical Critical Paths)& (IaaS Cloud Partial Critical Paths with Deadline Distribution), DCCP [32](Vahid A et al.) (Deadline Constrained Critical Path), Deadline-MDP(Deadline-Markov Decision Process) [33](Jia Y et al.), CD-PCP [34](Abrishami S et al.)(Cost-Driven Partial Critical Paths)etc., but these algorithms only consider task graph and server itself, which sort all task nodes and select the execution server prior to the actual scheduling. The above scheduling algorithms do not consider the change problem of sub-deadline and execution cost in the scheduling process. They do not consider the actual computation time (cost) on the execution server in the scheduling process.

The main contributions of this paper and a simple comparative analysis with reference [34] are summarized as follows: Reference [35] proposed a scheduling algorithm for cloud computing based on the driver of dynamic essential path, i.e., DDEP algorithm. This paper proposes a deadline-constrained task scheduling algorithm based on the analysis of the dynamic essential path from our previous work [35], i.e. Deadline-DDEP algorithm. The final objective is different between DDEP algorithm and Deadline-DDEP algorithm. The DDEP algorithm is to shorten the Makespan of task graph in the cloud computing. This paper proposes Deadline-DDEP algorithm to reduce the total execution cost while meeting the user’s deadline constraint. Our previous work (DDEP algorithm) uses the different priority values and the dynamic essential path values to confirm the scheduling order of all the task nodes. This paper proposes the dynamic sub-deadline strategy to compute the sub-deadline values for every task node based on our previous work. The strategy fully considers the dynamic sub-deadline affected by the dynamic essential path in the scheduling process. To the problem of selecting server for each task node, our previous work [34] uses the server that owns the earlier finish time to schedule task node. This paper propose the quality assessment of optimization cost strategy to solve the selective problem of scheduling server for all task nodes, the strategy selects the server that not only meets the sub-deadline but also owns the much lower execution cost. The experimental results show that, the proposed Deadline-DDEP produced remarkable performance improvement rate on the total execution cost while meeting the user’s deadline constraint.

## Related work

The heuristic algorithms for the deadline-constrained clouding computing scheduling problem have a common feature, which is the sub-deadline and scheduling result are done prior to the task actual scheduling. On the contrary, the proposed algorithm dynamically update the sub-deadline of task nodes in the actual scheduling process. The scheduling result is obtained when the task graph is fully completed. A simple comparative analysis of the proposed algorithm and the existing scheduling algorithms is as the following sections.

### (1) IC-PCP

IC-PCP (IaaS Cloud Partial Critical Paths) [31] computes EST(Earliest Start Time), EFT(Earliest Finish Time) and LFT(Latest Finish Time) for all task nodes, and then the task nodes are got in the PCPs(Partial Critical Paths). Firstly, schedule the unassigned task nodes without parent task nodes in the PCPs. If the current task node is finished before its Latest Finish Time, schedule it on the current cheapest server. Update the EST, EFT and LFT of all unassigned successor task nodes when the current task node is finished. The algorithm stops until there is no unassigned parent or child task node. The algorithm is simple and viable, and its time complexity is *O*(*n*^2^), where the number of task nodes is *n*.

Compute the EFT and LFT of the current task node by itself property and the minimum execution time of its successor task node by IC-PCP algorithm. The algorithm does not consider the EFT and LFT of the current task node. The actual execution time and communication time of its successor task nodes are affected the EFT and LFT of the current task node in the scheduling process. Compared with IC-PCP algorithm, the proposed algorithm dynamically update the sub-deadline by the deadline of task graph and the dynamic essential path of task node in the scheduling process. In this way, the time range of selecting the optimal server will be broaden. For the sort order of task nodes is obtained in the scheduling process by the proposed algorithm (Deadline-DDEP), which makes the sort order generated by Deadline-DDEP algorithm is more reasonable than IC-PCP algorithm.

### (2) DCCP

DCCP(Deadline Constrained Critical Paths) [32]algorithm is to first partition task graph into different levels based on their respective parallel and synchronization requirements. Compute the earliest finish time of all task nodes according to the average communication time and the minimum execution time. To the same level task nodes, their sub-deadline is equal to the maximum value of their earliest finish time. Obtain the CCPs (Constrained Critical Paths) task nodes according to their average execution time and communication time. All task nodes in a CCPs are executed on the same server that the cheapest server among servers and meet their sub-deadline. DCCP algorithm time complexity is *O*(*n*^2^ * *k*), where the number of task nodes is *n*; and *k* the number of server types.

DCCP algorithm selects all task nodes in a CCPs are executed in the server with the goal of avoiding communication time between task nodes, in this way, the choice of selecting cheaper server for a single task node is reduced, which may add to the total execution cost. Compared with the DCCP algorithm, the proposed algorithm uses the dynamic sub-deadline for each task node. It not only meets the deadline of task graph, but also adds to the choice of selecting cheaper server for each task node, and then minimizes the total execution cost.

### (3) Deadline-MDP

The main concept of Deadline-MDP (Deadline-Markov Decision Process) [33] algorithm divides task graph into many independent branches and synchronization tasks. Divide The overall deadline into sub-deadline for branches task according to their minimum processing time. The optimal decision is to minimize the execution cost of each branches task within the assigned sub-deadline. Because all parallel branches tasks own the same sub-deadline, to the multi-task-nodes and the longer execution path of branches tasks, which is executed on the faster and expensive server to meeting its sub-deadline, in this manner, the total execution cost may be increase. Compared with the Deadline-MDP, the proposed algorithm uses the dynamic sub-deadline according to the actual execution time and communication time, which adds to the choice that the optimal server.

### (4) CD-PCP

CD-PCP(Cost-driven Partial Critical Paths) [34] algorithm searches for the partial critical paths(PCP) according to the minimum execution time and minimum communication time. The task nodes in the PCP are scheduled within the user’s deadline firstly, the execution cost is minimized. The start time of task nodes in the PCP depends on the unscheduled parent task node. The unscheduled parent task node is executed on the better server while meeting its sub-deadline. This procedure continues recursively until all task nodes are scheduled successfully. Compared with the proposed algorithm uses the dynamic sub-deadline, the CD-PCP algorithm shorten the sub-deadline of unscheduled parent task nodes, which adds to the execution cost of unscheduled parent task nodes. Furthermore, the total execution cost may be increase.

## Data model

**Fig 1:**
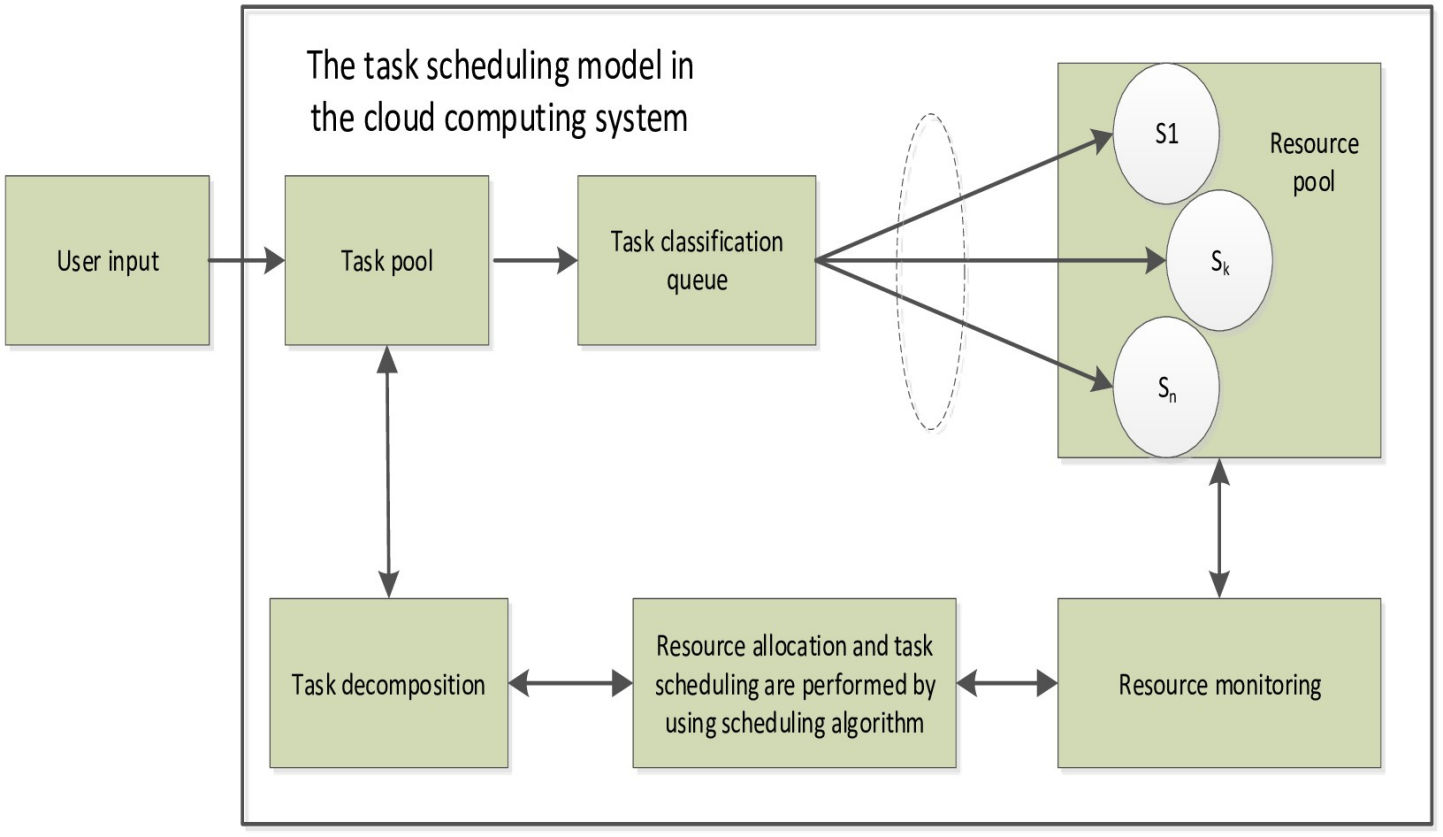
The task scheduling model in the cloud computing system.

The cloud computing system is a computer network composed of user, network and an easily extensible scheduling algorithm. The cloud providers offer the cloud computing resources and services to cloud users via the different scheduling algorithm. The target of cloud computing scheduling algorithm is how to map task to the corresponding server under meeting the user’s different QoS. Fig 1 shows the task-scheduling model in the cloud computing system.

The target of cloud computing scheduling algorithm is how to map task to the corresponding server under meeting the user’s different QoS. We first create the scheduling model by converting the cloud computing scheduling problem into the DAG scheduling problem [35]. The DAG graph is expressed: *G* = {*Q*, *E*, *S*}, where *Q* is the task node set of DAG graph, *Q* = {*Q*_*i*_, *Q*_2_, …, *Q*_*n*_}, *Q*_*i*_ represents the *ith* task node, *n* represents the number of task nodes; *E* is the set of communication costs among task nodes, *E* = {*e*_*ij*_}(*i*, *j* ∈ *Q*), and *e*_*ij*_ represents the precedence constraint relations such that *Q*_*i*_ should complete its execution before *Q*_*j*_ begins. *S* is the set of network servers, *S* = {*S*_1_, *S*_2_, …, *S*_*m*_}, *S*_*m*_ represents the *mth* server, *m* represents the number of servers, that is the processing machine of task node. *c* is the execution cost set of each server in each time interval, *c* = {*c*_1_, *c*_2_, …, *c*_*m*_}, *c*_*m*_ is the cost of each time interval of *mth* server.

The deadline-constrained DAG scheduling problem is described as follows: *D* represents the user’s deadline, *EST*(*Q*_*i*_, *S*_*m*_) represents the earliest start time for *Q*_*i*_ on the *S*_*m*_; and *EFT*(*Q*_*i*_, *S*_*m*_) represents the earliest finish time of *Q*_*i*_ on the *S*_*m*_. For the single entry task node *Q*_*i*_ on the *S*_*m*_:

$$EST\left(Q{}_{i},S{}_{m}\right)=T{}_{0}$$ (1)

$$EFT\left(Q{}_{i},S{}_{m}\right)=EST\left(Q{}_{i},S{}_{m}\right)+t{}_{i}{}_{m}$$ (2)

where *T*_0_ represents the application start time. For the other task nodes in the DAG graph:

$$EST\left(Q{}_{j},S{}_{m}\right)=\underset{Q_{i}\in pre\left(Q_{j}\right)}{\text{max}}\left(EFT\left(Q{}_{i},S{}_{n}\right)+e{}_{i}{}_{j}\right)+T{}_{0}$$ (3)

$$EFT\left(Q{}_{j},S{}_{m}\right)=t{}_{j}{}_{m}+EST\left(Q{}_{j},S{}_{m}\right)$$ (4)

where *Pre*(*j*)is the set of immediate predecessor task nodes of *Q*_*j*_. After all immediate predecessor task nodes of *Q*_*j*_ are finished, the data are transmitted to *Q*_*j*_; where *e*_*ij*_ represents the communication cost between *Q*_*i*_ and *Q*_*j*_. When all data required for *Q*_*j*_ have arrived, the server *S*_*m*_ begins to process *Q*_*j*_.

The objective functions of all task nodes on the DAG graph are described as:

$$Makespan=max(EFT\left(Q{}_{e}{}_{x}{}_{i}{}_{t}\right),S{}_{m})\leq D$$ (5)

$$Cost=\sum_{1\leq i\leq n}\left(t{}_{i}{}_{k}*c{}_{k}\right)$$ (6)

where *Q*_*exit*_ is a single exit task node, *t*_*ik*_ is the actual execution time of *Q*_*i*_ on the *S*_*k*_. The final objective is to minimize the total execution of task graph while meeting the user’s deadline, i.e., *min*(*Cost*) and *Makespan* ≤ *D*.

## Scheduling algorithm

The goal of scheduling algorithm is to minimize the execution cost of task graph while meeting the user-defined deadline. Whether the task graph will be finished within the user’s deadline depends on whether each task node will be finished in its sub-deadline. The dynamic essential path of task node is changeable constantly along with the actual execution time and communication time of its predecessor task node. The paper proposes the dynamical sub-deadline strategy based on the dynamic essential path changes of task node. The strategy fully considers the sub-deadline of task node affected by its dynamic essential path in the scheduling process. Under meeting the dynamic sub-deadline of task node, the quality assessment of optimization cost strategy is proposed. The strategy selects the relatively cheaper server to schedule each task node. Finally, the final objective of minimizing the total execution can achieve.

### Dynamic sub-deadline strategy

To explicitly describe the scheduling algorithm, we define the following terminology:

#### Dynamic essential path

Firstly, compute the path of task node based on the actual execution time of task node and the communication time with their predecessor task node. Because the path of task node will be changeable in the scheduling process, it is called as dynamic essential path(DEP).

In the cloud computing system, to the scheduling problem of a deadline-constrained DAG graph. For all task nodes, their dynamic essential path are got based on the actual execution time and the communication time with their predecessor task node. To the pre-scheduling task nodes, for their execution time and communication time with their predecessor task node are uncertainty, their dynamic essential paths will be changeable. The sub-deadline of taks node is associated with its dynamic essential path, so the sub-deadline is changeable. For the changeable sub-deadline, the dynamic sub-deadline strategy is proposed. The strategy will update the sub-deadline and sort order of the pre-scheduling task nodes according to their dynamic essential path. The concrete steps are as follows:

Step1 Initialize the dynamic essential path value for all task nodes. The path length values for all task nodes are obtained by the formula (7).
  $$DEP\left(Q{}_{j}\right)=\bar{t{}_{j}}+\underset{Q_{i}\in Pre\left(Q_{j}\right)}{\text{max}}\left(DEP\left(Q{}_{i}\right)+e{}_{i}{}_{j}\right)$$ (7)
  Where $\bar{t{}_{j}}$ is the average execution time of *Q*_*j*_, and *Pre*(*Q*_*j*_) is a set of the predecessor task nodes of *Q*_*j*_.
Step2 Search for the pre-scheduling task nodes. The entry task nodes have no predecessor task node in the DAG graph, compared with the other task nodes in the DAG graph, the entry task nodes are first pre-scheduling task nodes. The dynamic sub-deadline values of all entry task nodes are got firstly. The corresponding formula as follows:
  $$SD\left(Q{}_{e}{}_{n}{}_{t}{}_{r}{}_{j}\right)=\{\begin{array}{cc} \frac{\text{max}(EDP\left(Q{}_{e}{}_{n}{}_{t}{}_{r}{}_{j}\right))}{\text{max}(DEP\left(Q{}_{e}{}_{x}{}_{i}{}_{t}\right))}*D+(D-\text{max}\left(EDP\left(Q{}_{e}{}_{x}{}_{i}{}_{t}\right)\right) & D\geq \text{max}(EDP\left(Q{}_{e}{}_{x}{}_{i}{}_{t}\right))\\ \frac{\text{max}(EDP\left(Q{}_{e}{}_{n}{}_{t}{}_{r}{}_{j}\right))}{\text{max}(DEP\left(Q{}_{e}{}_{x}{}_{i}{}_{t}\right))}*D & D<\text{max}(EDP\left(Q{}_{e}{}_{x}{}_{i}{}_{t}\right)) \end{array}$$ (8)
  Where *Q*_*entrj*_ is an entry task node, *Q*_*exit*_ is an exit task node. Sort all entry task nodes in descending order by their dynamic essential path values. Firstly scheduled the entry task node that has longest dynamic essential path. Because its finish time influences indirectly the Makespan. Select the optimal servers for all entry task nodes by the quality assessment of optimization cost strategy in the above order list. Then update the dynamic essential path, execution time and execution server for all entry task nodes. The corresponding formula as follows:
  $$DEP\left(Q{}_{e}{}_{n}{}_{t}{}_{r}{}_{j}\right)=t{}_{e}{}_{n}{}_{t}{}_{r}{}_{y}{}_{k}$$ (9)
  Where *t*_*entryk*_ is the execution time of *Q*_*entrj*_ on the *S*_*k*_. When all entry task nodes have been finished, its successor task nodes are pre-scheduling task nodes. Update the dynamic essential path value of all pre-scheduling task nodes by formula (7). Compute the dynamic sub-deadline value of all pre-scheduling task nodes by the formula (10).
  $$SD\left(Q{}_{j}\right)=\{\begin{array}{cc} \frac{\underset{Q{}_{j}\in AllCurPre}{\text{max}}\left(EDP\left(Q{}_{j}\right)\right)}{\text{max}(DEP\left(Q{}_{e}{}_{x}{}_{i}{}_{t}\right))}*D+(D-\text{max}\left(EDP\left(Q{}_{e}{}_{x}{}_{i}{}_{t}\right)\right) & D\geq \text{max}(EDP\left(Q{}_{e}{}_{x}{}_{i}{}_{t}\right)))\\ \frac{\underset{Q{}_{j}\in AllCurPre}{\text{max}}\left(EDP\left(Q{}_{j}\right)\right)}{\text{max}(DEP\left(Q{}_{e}{}_{x}{}_{i}{}_{t}\right))}*D & D<\text{max}(EDP\left(Q{}_{e}{}_{x}{}_{i}{}_{t}\right))) \end{array}$$ (10)
  Where *AllCurPre* is the set of the pre-scheduling task nodes. Sort all pre-scheduling task nodes by their dynamic essential path value in descending order. Use the quality assessment of optimization cost strategy to schedule all pre-scheduling task nodes by their sort order and sub-deadline values. To accurately compute the dynamic essential path, the communication time (cost) is reduced to 0, i.e., *e*_*ij*_ = 0, when the two task nodes are scheduled on the same server, and *Q*_*i*_ is a predecessor task node of *Q*_*j*_. When all pre-scheduling task nodes have been finished, update their computation cost, processing servers and dynamic essential path values. The formula is as follow:
  $$DEP\left(Q{}_{j}\right)=t{}_{j}{}_{k}+\underset{Q_{i}\in Pre\left(Q_{j}\right)}{\text{max}}\left(DEP\left(Q{}_{i}\right)\right)+e{}_{i}{}_{j}$$ (11)
Step3 Schedule all exit task nodes. Define the dynamic sub-deadline value of all exit task nodes as *D* by the dynamic sub-deadline strategy. Update the dynamic essential path value of all exit task nodes by formula (7). Sort all exit task nodes by their dynamic essential path value in descending order. Use the quality assessment of optimization cost strategy schedule all exit task nodes by their sort order and dynamic sub-deadline values.

### Quality assessment of optimization cost strategy

Under meeting the dynamic sub-deadline value, this paper proposes the quality assessment of optimization cost strategy to solve the selective problem of scheduling server for all task nodes. The strategy considers a broader view of the total execution cost. The strategy selects the optimal server for each task node according to their sub-deadline, execution cost and finish time on each server, which makes the current task node and its successor task nodes to have the lower execution cost. The concrete steps as follows:

*Q*_*curr*_ represents the current task node. *SD*(*Q*_*curr*_) is defined as the dynamic sub-deadline of *Q*_*curr*_. *FT*_*Max*_(*Q*_*curr*_) and *FT*_*Min*_(*Q*_*curr*_) represents the maximum finish time and minimum finish time of *Q*_*curr*_ on all servers. *Cost*_*cheapest*_(*Q*_*curr*_) represents the cheapest execution cost of *Q*_*curr*_ on all servers. *Cost*_*Max*_(*Q*_*curr*_) and *Cost*_*Min*_(*Q*_*curr*_) represents the maximum execution cost and minimum execution cost of *Q*_*curr*_ on all servers. The time quality and cost quality of *Q*_*curr*_ on all servers is as follows:

$$TQ\left(Q{}_{c}{}_{u}{}_{r}{}_{r},S{}_{j}\right)=\frac{SD\left(Q{}_{c}{}_{u}{}_{r}{}_{r}\right)-FT\left(Q{}_{c}{}_{u}{}_{r}{}_{r},S{}_{j}\right)}{FT{}_{M}{}_{a}{}_{x}\left(Q{}_{c}{}_{u}{}_{r}{}_{r},S{}_{j}\right)-FT{}_{M}{}_{i}{}_{n}\left(Q{}_{c}{}_{u}{}_{r}{}_{r},S{}_{j}\right)}$$ (12)

$$CQ\left(Q{}_{c}{}_{u}{}_{r}{}_{r},S{}_{j}\right)=\frac{Cost\left(Q{}_{c}{}_{u}{}_{r}{}_{r},,S{}_{j}\right)-Cost{}_{c}{}_{h}{}_{e}{}_{a}{}_{p}{}_{e}{}_{s}{}_{t}\left(Q{}_{c}{}_{u}{}_{r}{}_{r}\right)}{Cost{}_{M}{}_{a}{}_{x}\left(Q{}_{c}{}_{u}{}_{r}{}_{r},S{}_{j}\right)-Cost{}_{M}{}_{i}{}_{n}\left(Q{}_{c}{}_{u}{}_{r}{}_{r},S{}_{j}\right)}$$ (13)

Where *TQ*(*Q*_*curr*_, *S*_*j*_) measures how much closer to the dynamic sub-deadline and the finish time of *Q*_*curr*_ on the *S*_*j*_, i.e., measures the finish time urgency of *Q*_*curr*_. When the *TQ*(*Q*_*curr*_, *S*_*j*_) value is negative number, it means *Q*_*curr*_ is not finished within its dynamic sub-deadline on the *S*_*j*_, then *Q*_*curr*_ rejects to be scheduled on the *S*_*j*_. When *TQ*(*Q*_*curr*_, *S*_*j*_) is a bigger positive number, it means that the finish time of *Q*_*curr*_ is farther its dynamic sub-deadline on the *S*_*j*_. When *TQ*(*Q*_*curr*_, *S*_*j*_) is a smaller positive number, the finish time of *Q*_*curr*_ is closer to its dynamic sub-deadline on the *S*_*j*_. *CQ*(*Q*_*curr*_, *S*_*j*_) measures how much less the execution cost of *Q*_*curr*_ on the *S*_*j*_ than the cheapest execution cost on all servers, which is used to avoid selecting the server that has worse performance and higher execution cost. *QM*(*Q*_*curr*_, *S*_*j*_) is defined to select the better reasonable server for *Q*_*curr*_, which is used to select the server that has not only lower execution cost, but also meets its dynamic sub-deadline. When the *QM*(*Q*_*curr*_, *S*_*j*_) is bigger value, it means the finish time of *Q*_*curr*_ on the *S*_*j*_ is farther than its dynamic sub-deadline. Its execution cost on the *S*_*j*_ is closer to the cheapest execution cost, in contrast, it means the finish time of *Q*_*curr*_ on the *S*_*j*_ is farther than its dynamic sub-deadline, and its execution cost on the *S*_*j*_ is larger than the cheapest execution cost. *QM*(*Q*_*curr*_, *S*_*j*_) formula is as follows:

$$QM\left(Q{}_{c}{}_{u}{}_{r}{}_{r},S{}_{j}\right)=TQ\left(Q{}_{c}{}_{u}{}_{r}{}_{r},S{}_{j}\right)+CQ\left(Q{}_{c}{}_{u}{}_{r}{}_{r},S{}_{j}\right)$$ (14)

The quality assessment of optimization cost strategy selects the server that has smaller *QM*(*Q*_*curr*_, *S*_*j*_) value to schedule *Q*_*curr*_.

**Fig 2:**
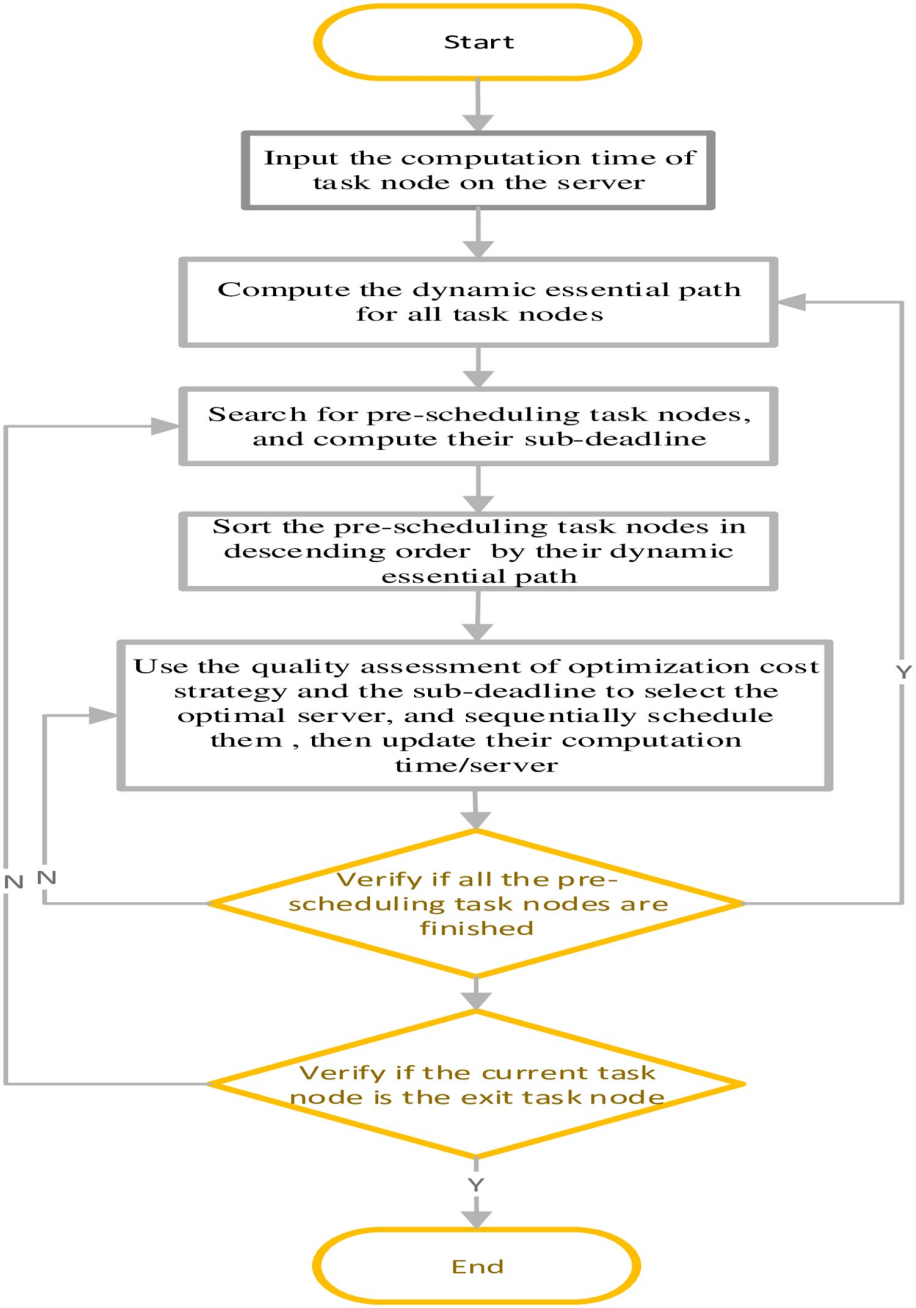
The flowchart of Deadline-DDEP algorithm.

To the reader understand the proposed scheduling algorithm clearly, we draw the flowchart of the proposed algorithm. Shown in Fig 2.

### An illustrative example

**Fig 3:**
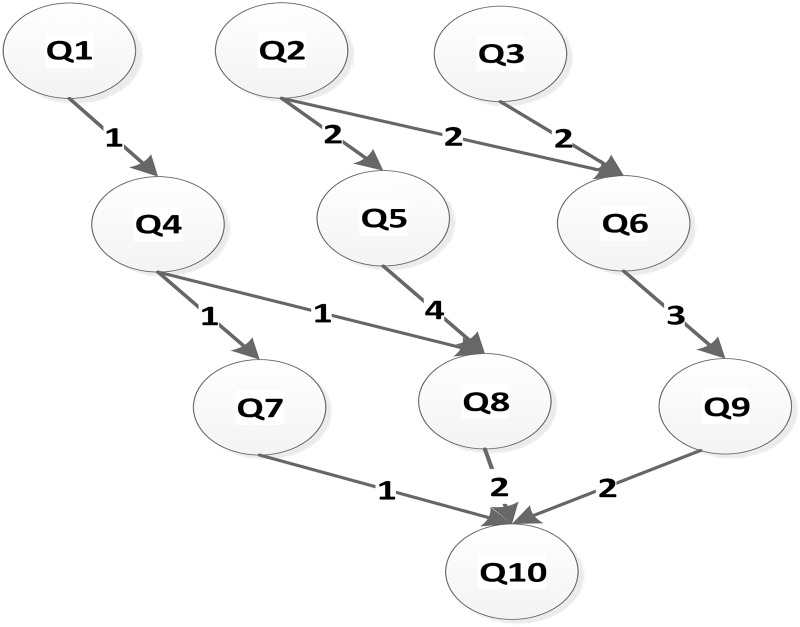
An application DAG Graph.

This paper converts a workflow into the DAG graph shown in Fig 3. The computation time on the three different types (heterogeneous) server are also given in the Table 1. It is assumed that three types server (S1, S2, S3) are used to schedule the DAG graph, and all servers are connected with communication links of the same capacity. There are many same type servers. Thus, the communication time between task nodes is determined by the edge of the DAG graph shown in Fig 3. The time interval of the computation server is assumed to be 10. The unit price of S1, S2, S3 is 5, 2, 1 respectively. The Deadline of a workflow in Fig 3 is 40 unit time. We demonstrate the implementation process of Deadline-DDEP algorithm.

**Table 1:** The computation time of the task graph on the different server in Fig 3.

Task Node	S1	S2	S3
Q1	2	6	8
Q2	5	13	16
Q3	3	5	9
Q4	5	7	10
Q5	3	8	11
Q6	4	6	11
Q7	5	8	11
Q8	3	6	8
Q9	5	8	14
Q10	5	10	16

This paper converts a workflow into the DAG graph shown in Fig 3. The computation time on the three different types (heterogeneous) server are also given in Table 1. It is assumed that three type servers (S1, S2, S3) are used to schedule the DAG graph, and all servers are connected with communication links of the same capacity. There are many same type servers. The communication time between task nodes is denoted by the edge of the DAG graph shown in Fig 3. The unit price of S1, S2, S3 is 5, 2, 1 respectively. The Deadline of a workflow in Fig 3 is 40 unit time. We demonstrate the implementation process of Deadline-DDEP algorithm.

DDEP algorithm is a scheduling algorithm for a deadline-constrained workflow in the cloud computing system and contains four major data phases: (1)The computation time phase, (2) the communication time phase, (3) the dynamic essential path phase, and (4)the pre-scheduling task node phase.

1. Computation time phase and communication time phase
  The two phases are an original array shown in Fig 3. The workflow owns the phase or table that stores the computation time of each task node on the different servers. The communication time between task nodes is stored by the adjacent matrix.
2. The dynamic essential path and the pre-scheduling task node phase
  The dynamic essential path phase stores the dynamic essential path for all task nodes. The QM value, execution time and scheduling server of all pre-scheduling task nodes are stored in the pre-scheduling task nodes phase. The implementation process of task graph in Fig 3 as follow:
  Step1 Initialize the dynamic essential path for all task nodes. Compute the dynamic essential path values for all task nodes by the formula (7). As shown in Table 2.
  Step2 Chedule all entry task nodes. Compared with the other task nodes, all entry task nodes first become the pre-scheduling task nodes. Q1, Q2 and Q3 will turn to the pre-scheduling task node firstly. According to the dynamic essential path value of Q1, Q2 and Q3 in the Step1, sort Q1, Q2 and Q3 in descending order: Q2, Q3 and Q1. Compute the dynamic sub-deadline and QM value for Q1, Q2 and Q3 by formula (8), (12)–(14). The related values show in the Table 1. If the finish time of *Q*_*i*_ is greater than its dynamic sub-deadline on the *S*_*j*_, the *QM*(*Q*_*i*_, *S*_*j*_) value is set to infinity by the Deadline-DDEP algorithm. Select the server that has the smaller QM value to schedule each entry task node. The corresponding value shows in the Table 2.
  Step3 Update the dynamic essential path for each task node. When Q1, Q2 and Q3 have been finished, their execution time and execution server are updated shown in the Table 2. Update the dynamic essential path of Q1, Q2 and Q3 to 2, 5 and 3 by formal (9). Update the dynamic essential path of other unscheduled task nodes by formula (7).
  Step4 Update the pre-scheduling task nodes phase. After all entry task nodes are finished, their successor task nodes become the pre-scheduling task node. When Q1, Q2 and Q3 have been finished, Q4, Q5 and Q6 turn to be pre-scheduling task nodes. According to the dynamic essential path value of Q4, Q5 and Q6 in the Step3, sort Q4, Q5 and Q6 in descending order: Q5, Q6 and Q4. Compute the dynamic sub-deadline and QM of Q4, Q5 and Q6 by formula (10), (12)–(14). Select the server that has smaller QM to schedule each pre-scheduling task node. The corresponding values show in the Tables 2 and 3.
  Step5 Update the dynamic essential path for each task node. When Q4, Q5 and Q6 have been finished, their execution time and execution server are updated to the values shown in the Tables 2 and 3. The dynamic essential path of Q4, Q5 and Q6 is updated to 7, 15, 13 by formula (11). Update the dynamic essential path of other unscheduled task nodes by formula (7).
  Step6 Scheduling all exit task nodes. The dynamic sub-deadline of all exit task nodes is 40. Compute the QM values of all exit task nodes by formula (14). Select the server that has smaller QM value to schedule each pre-scheduling task node. The corresponding values show in the Tables 2 and 3. The total execution cost of task graph in the Fig 3 shows in the Table 3.

**Table 2:** The values of each parameter for each step of running proposed algorithm on the DAG graph of Fig 3.

		Q1	Q2	Q3	Q4	Q5
Step1	Initialize EP	5.33	11.33	5.67	12.66	20.66
Step2	the state of task node	Pre	Pre	Pre	/	/
	Sub-deadline	10.22	10.22	10.22	/	/
	QM value	S1.1: 0.055	S1.1: 0	S1.1: 0.555	/	/
	QM value	S2.1: 2.055	S2.1: ∞	S2.1: 2.305	/	/
	QM value	S3.1: 1.055	S3.1: ∞	S3.1: 0.805	/	/
	Selected server	S1.1	S1.1	S1.1	/	/
Step3	Update EDP	2	5	3	10.33	14.33
Step4	the state of task node	Finished	Finished	Finished	Pre	Pre
	Sub-deadline	10.1	10.1	10.1	16.62	16.62
	QM value	S1.1: 0.055	S1.1: 0	S1.1: 0.555	S1.1: 1.27	S1.1: 1.724
	QM value	S2.1: 2.055	S2.1: ∞	S2.1: 2.305	S2: ∞	S2.1: 0.99
	QM value	S2.2: /	S2.2:/	S2.2:/	S2.2: ∞	S2.2: /
	QM value	S3.1: 1.055	S3.1: ∞	S3.1: 0.805	S3: ∞	S3.1: ∞
	Selected server	S1.1	S1.1	S1.1	S1.1	S2.1
Step5	Update EDP	2	5	3	7	15
Step6	the state of task node	Finished	Finished	Finished	Finished	Finished
	Sub-deadline	10.1	10.1	10.1	16.62	16.62
	QM value	S1.1: 0.055	S1.1: 0	S1.1: 0.555	S1.1: 1.27	S1.1: 1.724
	QM value	S2.1: 2.055	S2.1: ∞	S2.1: 2.305	S2.1: ∞	S2.1: 0.99
	QM value	S2.2: /	S2.2:/	S2.2:/	S2.2: ∞	S2.2: /
	QM value	S3.1: 1.055	S3.1: ∞	S3.1: 0.805	S3.1: ∞	S3.1: ∞
	Selected server	S1.1	S1.1	S1.1	S1.1	S2.1
		Q6	Q7	Q8	Q9	Q10
Step1	Initialize EP	20.33	22.66	30.33	32.33	44.33
Step2	the state of task node	/	/	/	/	/
	Sub-deadline	/	/	/	/	/
	QM value	/	/	/	/	/
	QM value	/	/	/	/	/
	QM value	/	/	/	/	/
	Selected server	/	/	/	/	/
Step3	Update EDP	14	20.33	24	26	38.33
Step4	the state of task node	Pre	/	/	/	/
	Sub-deadline	16.62	/	/	/	/
	QM value	S1.1: 1.37	/	/	/	/
	QM value	S2.1: ∞	/	/	/	/
	QM value	S2.2: 0.34	/	/	/	/
	QM value	S3.1: ∞	/	/	/	/
	Selected server	S2.2	/	/	/	/
Step5	Update EDP	13	17	24.67	19	37
Step6	the state of task node	Finished	Pre	Pre	Pre	/
	Sub-deadline	16.62	29.67	29.67	29.67	40
	QM value	S1.1: 1.37	S1.1: 1.21	S1.1: 2.53	S1.1: 1.63	S1.1: 1.67
	QM value	S2.1: ∞	S2.1: 1.58	S2: 1.53	S2.1: 0.82	S2.1: ∞
	QM value	S2.2: 0.34	S2.2: ∞	S2.2: 1.53	S2.2: 0.63	S2.2: 0.22
	QM value	S3.1: ∞	S3.1: 1.21	S3.1: 0.53	S3: ∞	S3.1: ∞
	Selected server	S2.2	S1.1	S3.1	S2.2	S2.2

**Table 3:** Server which are launched by Deadline-DDEP to execute the task graph in Fig 3.

Server	Start time	Stop time	Total cost	Assigned task nodes
S1.1	0	20	10	Q2, Q3, Q1, Q4, Q7
S2.1	7	16	4	Q5
S2.2	10	39	6	Q6, Q9, Q10
S3.1	19	27	2	Q8

Table 2 shows the values parameter for each step of running proposed algorithm in the rows. The states of task node are “Pre-scheduling”, “Finished”. “Pre-scheduling” is that the predecessor task nodes of the current task node are finished, the current task node is a schedulable task node. “Finished” is that the current task node has been executed. If the QM value of server is infinite, the task node is not executed on the server. Table 3 shows the “Start time”, “End time” and “Total cost” of every server.

### Complexity analysis

Time complexity is the amount of computation required to execute the algorithm. The time complexity of Deadline-DDEP algorithm contains two separate components: one is the time complexity of the sub-deadline strategy, and the other is the time complexity of the quality assessment of optimization cost strategy. It is assumed that *k* is the number of task nodes, and *n* is the number of server types. The specific time complexity analysis is as follows.

1. The time complexity of the dynamic sub-deadline strategy contains three separate components. The adjacent matrix is used to store the relationships (communication time) between task nodes in the task graph. The number of task nodes is *n*, and the size of adjacent matrix is *n* * *n*. First part is the number of searching for the pre-scheduling task node is *n*. Second part is the number of computing the dynamic essential path of all task nodes. Because the maximum number of the predecessor task nodes of the current task node is *n*, the maximum number of computing the dynamic essential path of the current task node is *n*; the maximum number of computing the dynamic essential path of all task nodes is *n* * *n*. Third part is the number of computing the dynamic sub-deadline for all task nodes, whose maximum number is *n*. The maximum number of computing the sub-deadline of all task nodes is *n* + *n* * *n* + *n* = *n*^2^ + 2 * *n*, the time complexity of the dynamic sub-deadline strategy is *O*(*n*^2^).

2. The time complexity of the quality assessment of optimization cost strategy contains two separate components. First part is the time complexity of sorting all task nodes by the dynamic essential path. Sort all task nodes in descending order by their dynamic essential path, whose time complexity is *O*(*n* log *n*). Second part is the scheduling server of all task nodes are got according to their sort order and QM values, the maximum number of computing QM values for all task nodes is *k* * *n*, where *k* is the number of server types, *n* is far greater than *k*, its time complexity is *O*(*k* * *n*) = *O*(*n*). The time complexity of the quality assessment of optimization cost strategy is *O*(*n* log *n*) + *O*(*n*).

To summarize, the time complexity of the proposed algorithm is *O*(*n*^2^) + *O*(*n* log *n*) + *O*(*n*), approximated as *O*(*n*^2^).

## Experiment result and comparison

In this section, we present simulation experiments on the Deadline-DDEP algorithm. The paper uses the different types sample task graphs to evaluate the performance of proposed algorithm. There are two ways to choose the sample task graph. One is using a random DAG generator to create the different structure task graph, other is using a library of realistic task graph to obtain the different type task graph. Although the latter seems to be a better choice, unfortunately, there is no such a comprehensive library available to researchers. We designed a random generator to ensure the accuracy of the simulation experiments, and used IC-PCP, DCCP and CD-PCP algorithms in benchmark experiments to obtain a relatively objective evaluation. The experimental model is a rather typical computing model-DAG scheduling model. The simulation experiments are as follows. First of all, the experiment environment is introduced. Secondly, the experimental parameters are presented. Thirdly, the performance results are covered.

### Experimental environment

Experimental platform is Win8 64 bit, Matlab2012, CPU: intel i5, Memory:8G. The generator depended on several input parameters according to user requirements. The corresponding input parameters are listed in Table 4.

**Table 4:** User parameters.

Parameters	User defined values
n	Number of task nodes
m	Number of task servers
[MinComputTime, MaxComputTime]	The computation time rang of task nodes.
[MinComuniTime, MaxComuniTime]	The comunication time range of task nodes.
[0, MaxOutDegree]	The out-degree range of task node.
[0, MaxInDegree]	The in-degree range of task node.
*α*_*d*_	The deadline factor of task graph is [0, 1]
*c*(*S*_*k*_)	The unit price of each server, is got by formula (19)

The following experiment results were acquired, as generated with scheduling of the randomly generated DAG graph using IC-PCP, DCCP and CD-PCP algorithms.

### Experiment parameters

The parameters about the deadline and cost are by definition in our experiment to evaluate the performance of the proposed algorithm. They are associated with the scheduling result of task graph. The deadline parameter of task graph is *D*, to specifically define *D*, we first define the following parameters: *CPFT*_*max*_ and *CPFT*_*min*_, which represents the maximum and minimum finish time of all task nodes in the critical path. The corresponding formula as follows:

$$CPFT{}_{m}{}_{i}{}_{n}=\sum_{Q_{i},Q_{j}\in CP\&Q_{j}\in CriPre\left(Q_{i}\right)}\left(t{}_{m}{}_{i}{}_{n}\left(Q{}_{i}\right)+e{}_{i}{}_{j}\right)$$ (15)

$$CPFT{}_{m}{}_{a}{}_{x}=\sum_{Q_{i},Q_{j}\in CP\&Q_{j}\in CriPre\left(Q_{i}\right)}\left(t{}_{m}{}_{a}{}_{x}\left(Q{}_{i}\right)+e{}_{i}{}_{j}\right)$$ (16)

Where is the set of all task nodes in the critical path, *CriPre*(*Q*_*i*_) is the set of all predecessor task nodes of *Q*_*i*_ in the critical path, *t*_*min*_(*Q*_*i*_) and *t*_*max*_(*Q*_*i*_) represent the maximum and minimum execution time of *Q*_*i*_ on all servers, i.e., the fastest and lowest execution time. Because the finish time of all task nodes in the critical path indirectly influence the completion time of task graph, so the deadline of task graph is defined according to the *CPFT*_*max*_ and *CPFT*_*min*_ parameter. The corresponding formula as follows:

$$D=CPFT{}_{m}{}_{i}{}_{n}+\alpha {}_{d}*\left(CPFT{}_{m}{}_{a}{}_{x}-CPFT{}_{m}{}_{i}{}_{n}\right)$$ (17)

The total execution cost of task graph is associated to the execution cost of each task node. The execution cost of task node is associated to the execution time and the unit price of server, so the unit price of *S*_*k*_, *S*_*k*_ ∈ *S*, that is used to the experiment is defined as follows:

$$c\left(S{}_{k}\right)=\beta {}_{S}{}_{k}*\left(\beta {}_{S}{}_{k}+1\right)/2$$ (18)

where *β*_*Sk*_ represents the ratio of the CPU processing capacity to that of the fastest server of *S*_*k*_. The unit price of all servers will be in the range of [0, 1]. The unit price of the fastest server is 1. There are five types server in our experiment, and whose CPU number is 2, 4, 8, 16, 32, respectively. The unit price of all servers is 17/512, 9/128, 5/32, 3/8, 1, respectively.

### Performance metrics analysis

**Fig 4:**
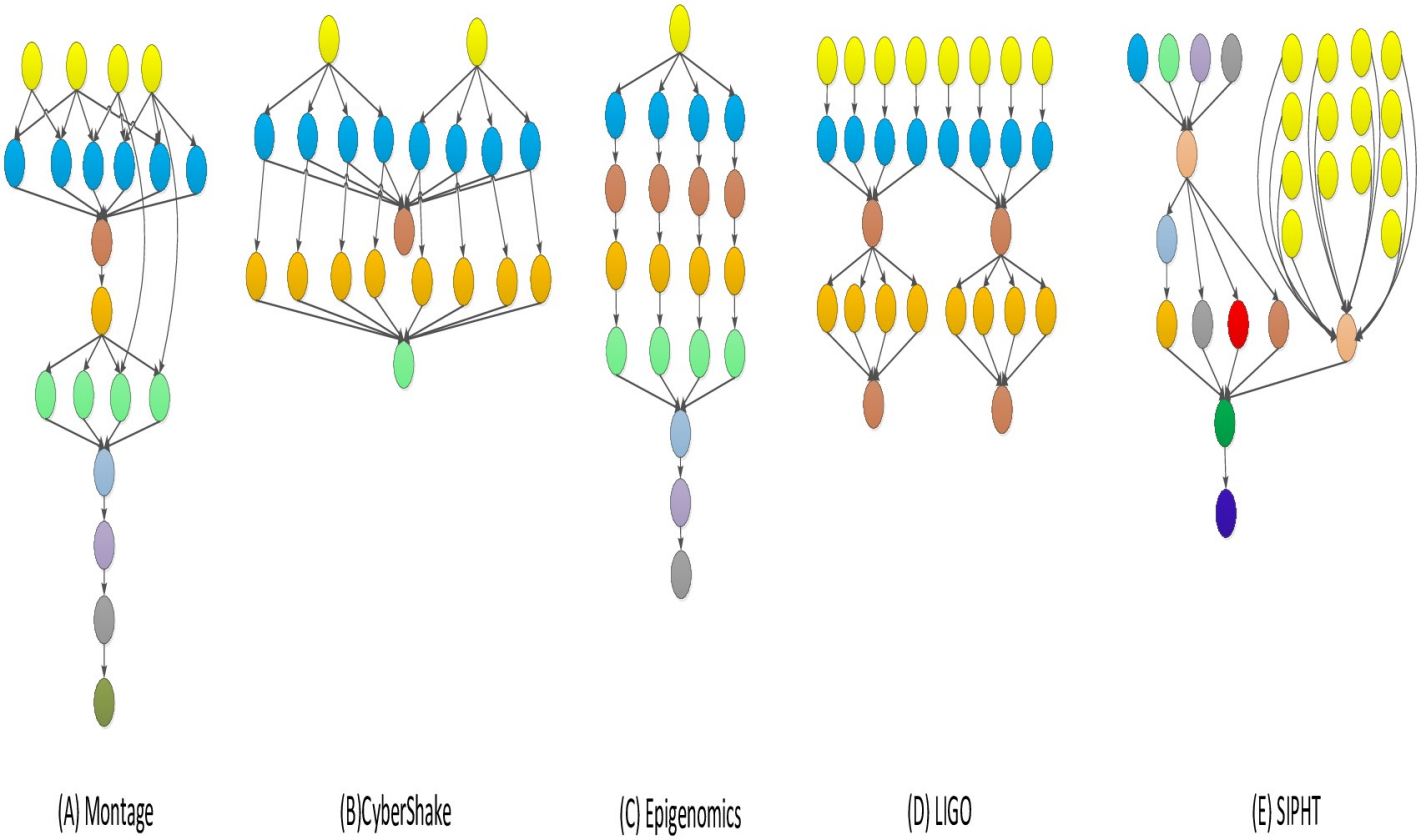
The structure of five realistic scientific workflows from reference [30].

This section shows the scheduling result analysis of the different structure DAG graph, Bharathi et al. [36] proposes the structure of five realistic task graph: Montage, CyberShake, Epigenomics, LIGO and SIPHT, shown in Fig 4. To evaluate the performance of the proposed algorithm, we adopt the common performance comparison metrics *NC*(*NormalizedCost*) and *PSR* (*PlanningSuccessfulRate*). *NC* is the main performance measure for a scheduling algorithm on a graph and is the ratio of the total execution cost to the cheapest execution cost of task graph with a formula defined by:

$$NC=\frac{TSC}{C{}_{c}{}_{h}{}_{e}{}_{a}{}_{p}{}_{e}{}_{s}{}_{t}}$$ (19)

where *C*_*cheapest*_ is the execution cost that all task nodes are executed on the cheapest server. If the *NC* value is smaller, the algorithm performance is better; if the algorithm performance is worse, the *NC* value is larger. The average *NC* values over several DAG graphs are used to our experiment.

*PSR* is the ratio of the successful scheduling number of task graph to the total number of the experimental task graph. The *PSR* formula is by definition:

$$PSR=100*\frac{SuccesfulPlanningNumber}{TotalNumberInExperiment}$$ (20)

Where *SuccesfulPlanningNumber* is the successful scheduling number of task graph under meeting the defined deadline. If the SPR value is smaller, the algorithm performance is worse, whereas if the SPR value is larger, the algorithm performance is better. The average SPR values over several DAG graphs are used to our experiment.

**Fig 5:**
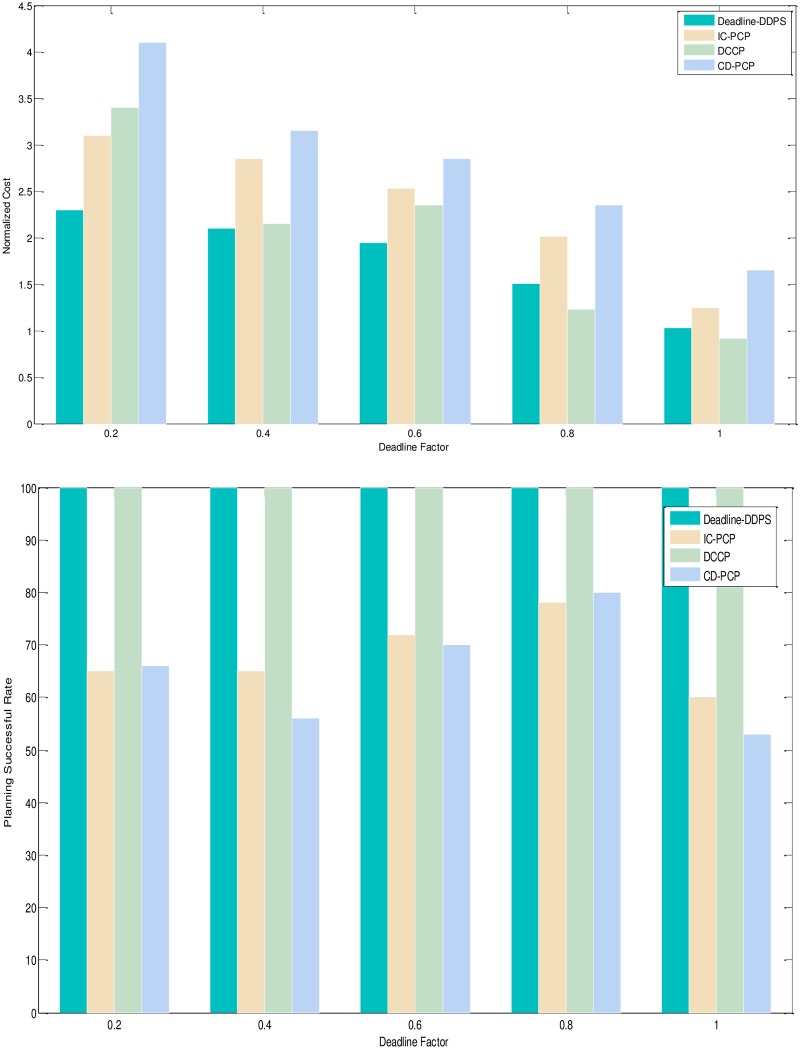
NC and PSR with Montage structure graph.

**Fig 6:**
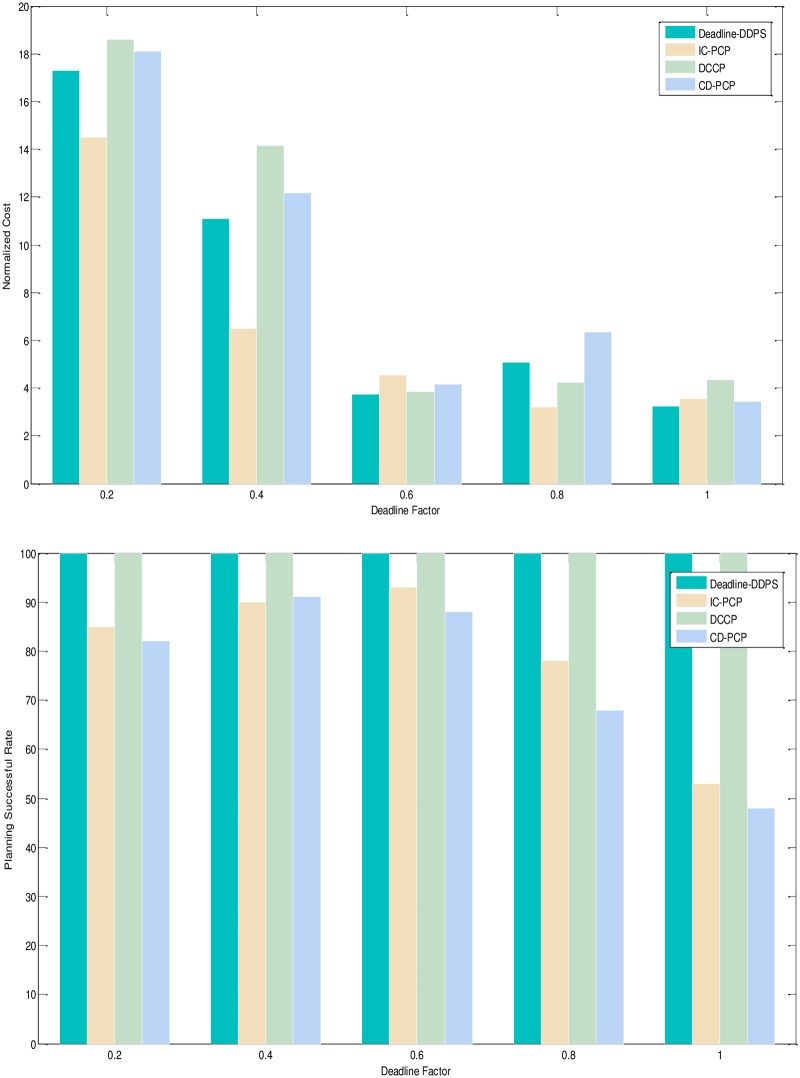
NC and PSR with CyberShake structure graph.

**Fig 7:**
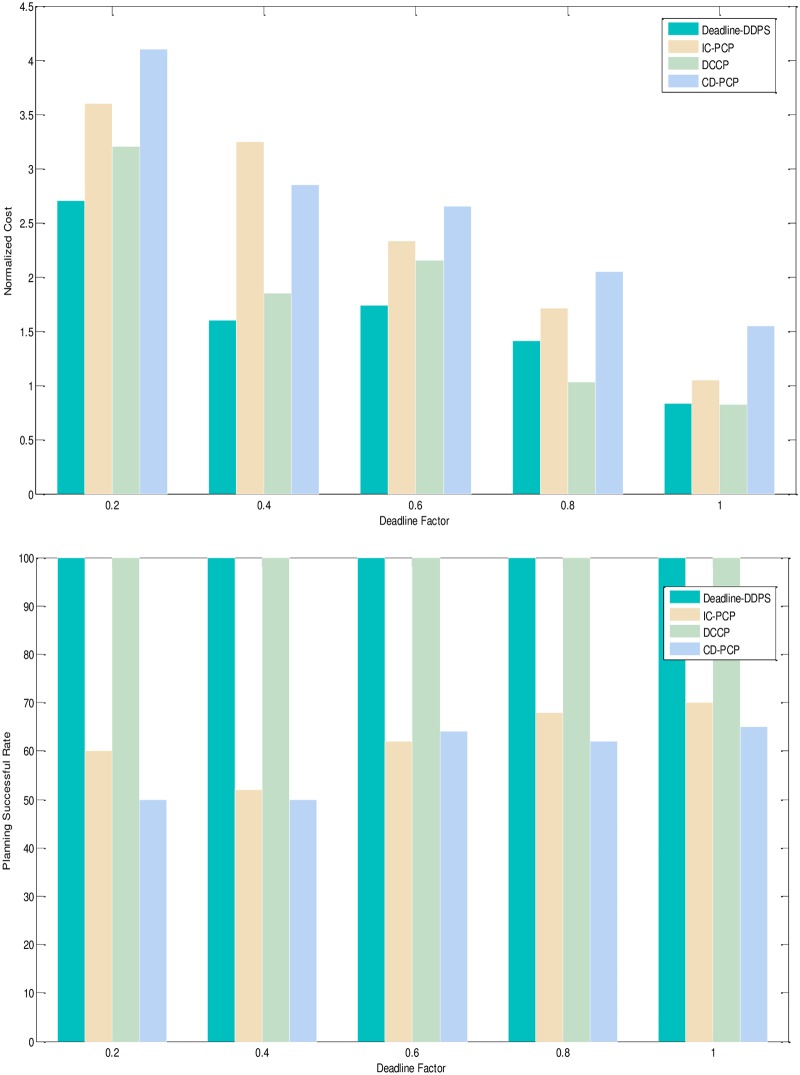
NC and PSR with Epigenomics structure graph.

**Fig 8:**
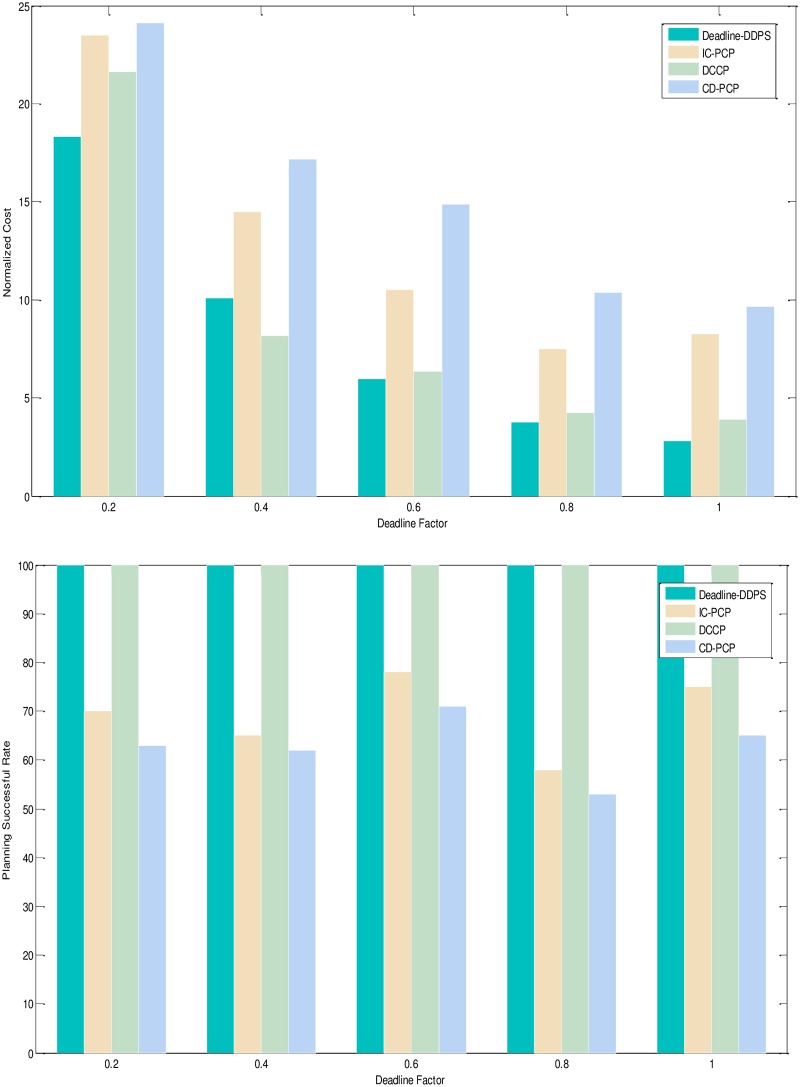
NC and PSR with LIGO structure graph.

**Fig 9:**
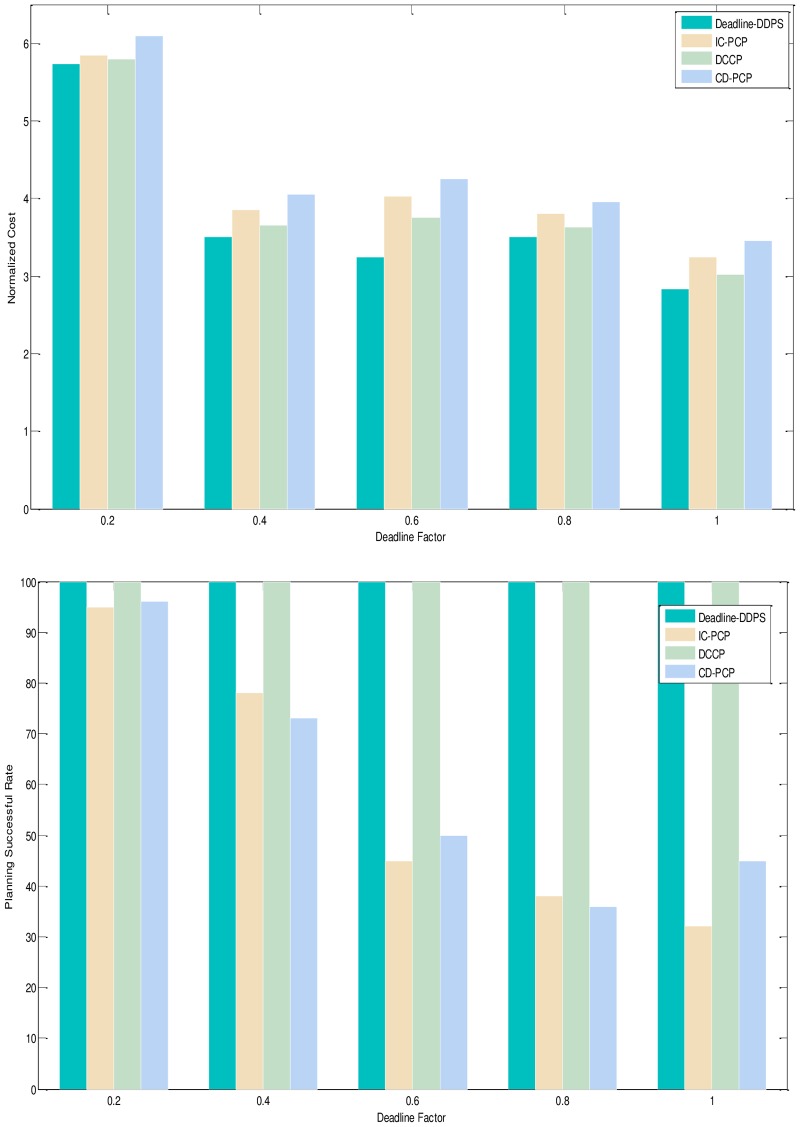
NC and PSR with SIPHT structure graph.

**Fig 10:**
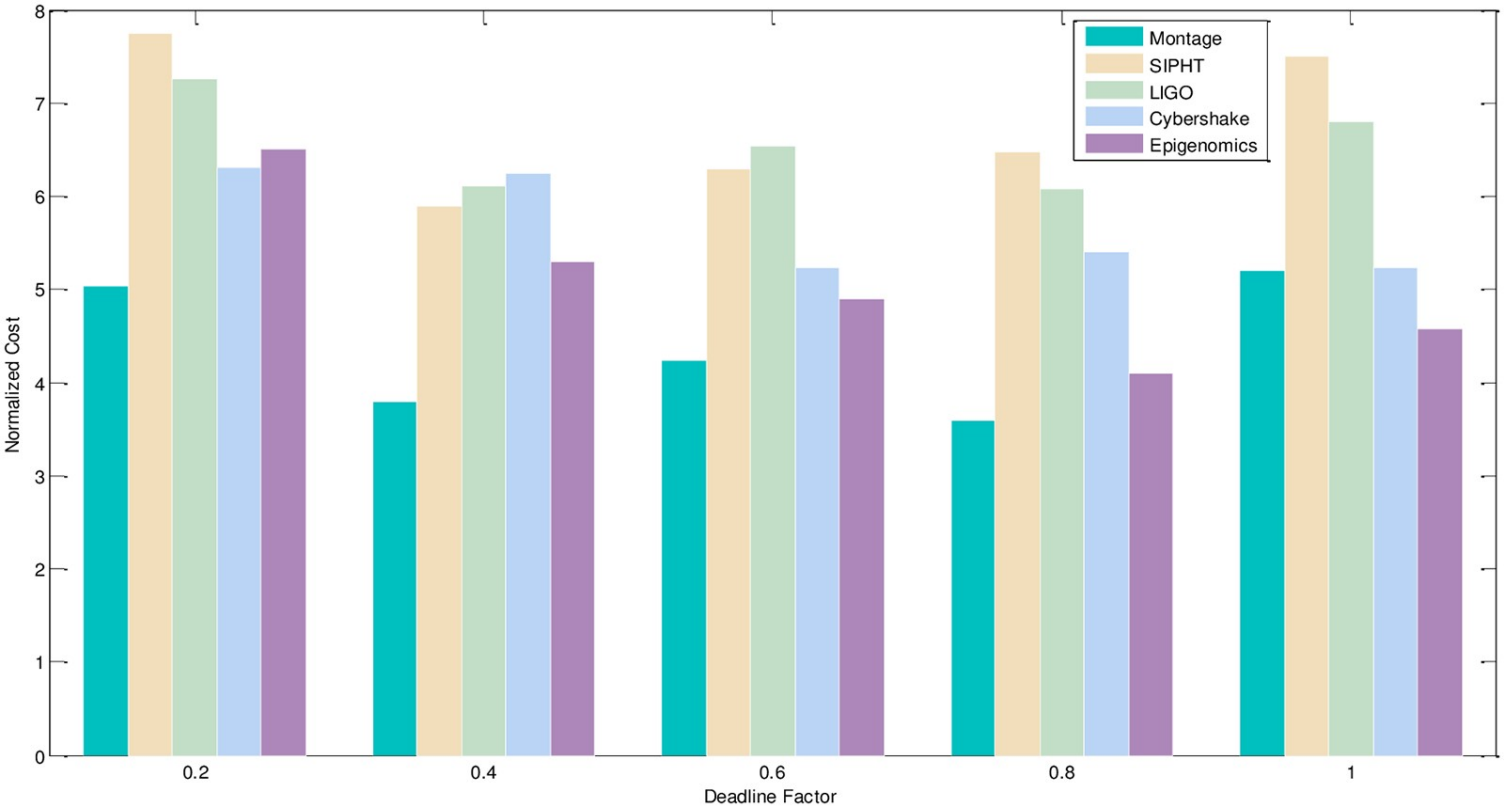
NC and PSR of small size DAG graph by the Deadline-DDEP algorithm.

**Fig 11:**
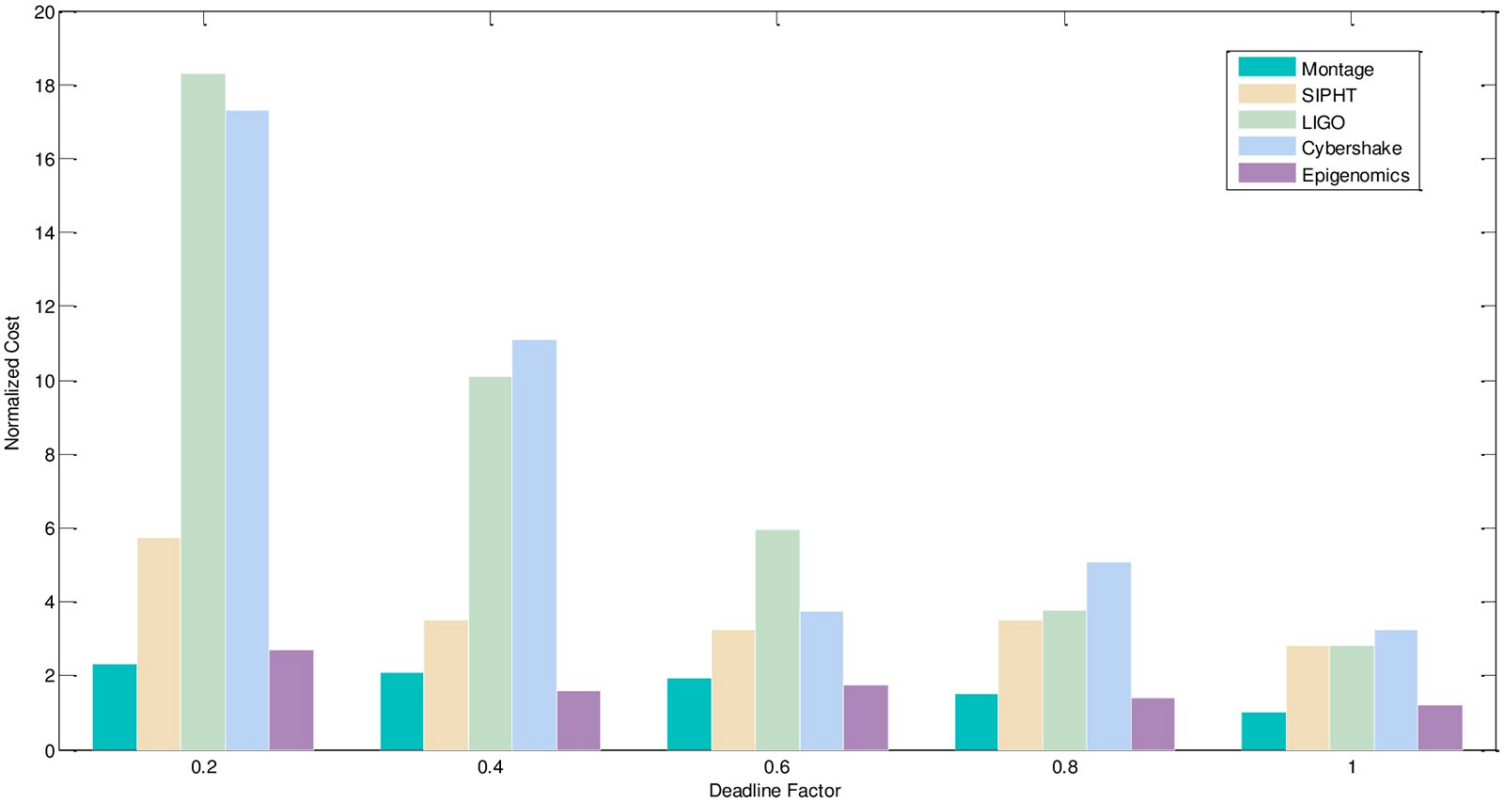
NC and PSR of medium size DAG graph by the Deadline-DDEP algorithm.

**Fig 12:**
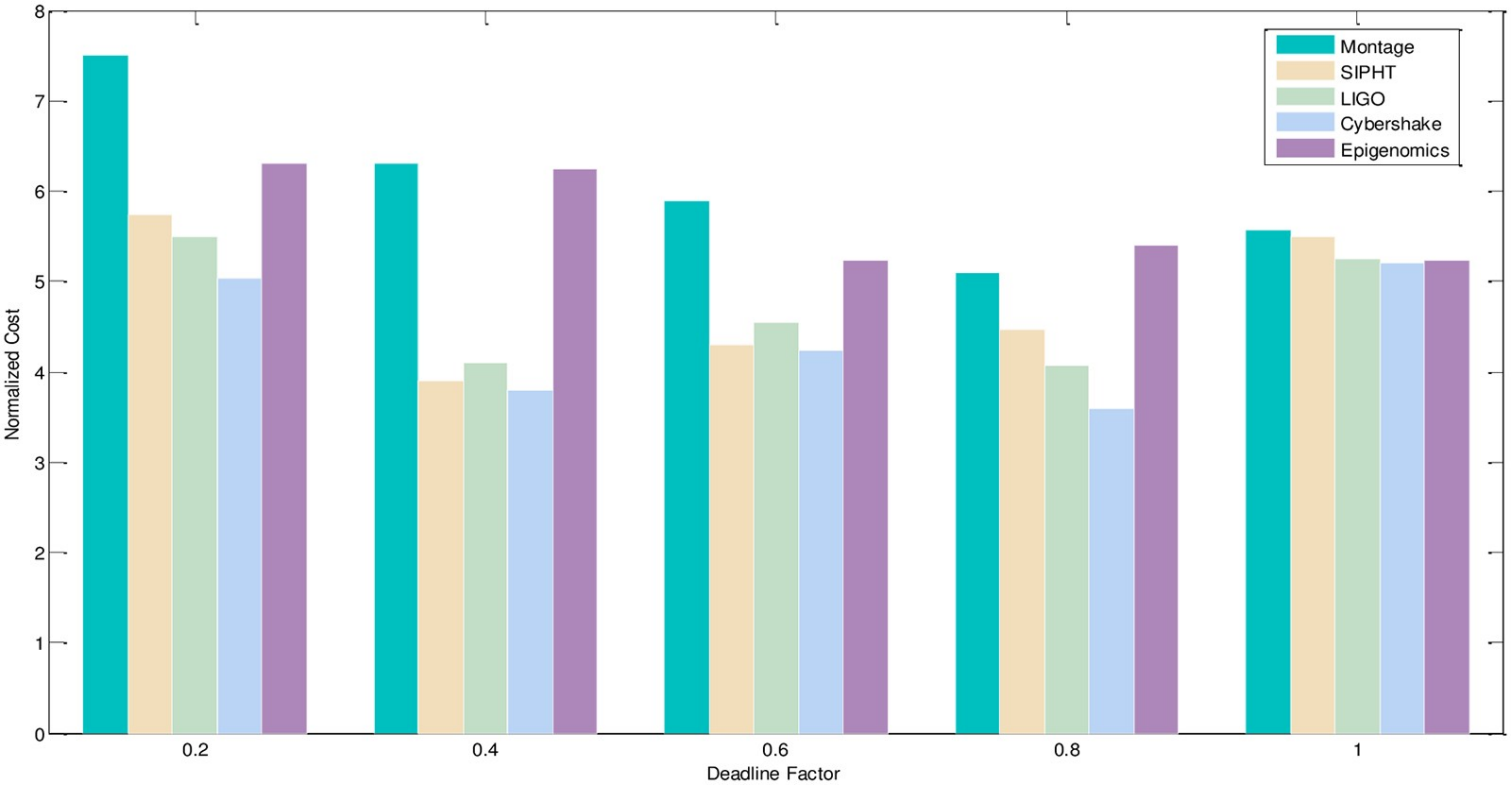
NC and PSR of large size DAG graph by the Deadline-DDEP algorithm.

1) Experimental analysis of the task graph structure. The goal is to verify the influence of the task graph structure on the scheduling algorithm by the NC and PSR. To show the performance of the proposed algorithm, we adopt different structure, different deadline-constrained and same size of DAG graph that are scheduled on the same-size type server to obtain the experimental result. We set the size of task graph to 100, and the number of server type to 5. The computation time is generated randomly in the [5, 10]. The communication time is generated randomly in the [5, 10]. The out-degree and in-degree of task graph are also randomly generated in the [1, 10]. The deadline factor of task graph is set to {0.2, 0.4, 0.6, 0.8, 1.0}. Figs 5–9 shows the obtained comparative results for average NC and average SPR of Montage, CyberShake, Epigenomics, LIGO and SIPHT by the different algorithm, as averaged over 100 runs for the same-deadline-factor task graph. According to the contrast analysis of the experimental result in Figs 5–9, the average NC of Deadline-DDEP algorithm is better than those of IC-PCP algorithm, DCCP algorithm and CD-PCP algorithm by 10.3%, 18.3% and 30.8%, respectively. Fig 6 shows that the value of average NC by the Deadline-DDEP algorithm is higher than by IC-PCP algorithm, but is lower than by DCCP and IC-PCP algorithm. It is because the Deadline-DDEP algorithm selects the faster CPU and higher price of server to schedule the task graph that has the same dynamic sub-deadline and the multi-parallel task nodes. The average PSR of Figs 5–9 show all experimental task graphs are successfully finished in the defined deadline by the Deadline-DDEP and DCCP algorithm, but the IC-PCP and CD-PCP have higher failure rate. This is because the Deadline-DDEP algorithm gets the dynamic sub-deadline for every task node by its dynamic essential path and the deadline of task graph.

2) Experimental analysis of the task graph scale. The goal is to verify the influence of the different size and different deadline-constrained task graph on the scheduling algorithm by the NC and PSR. We adopt the different size, different deadline-constrained and different structure of DAG graph and schedule them on same type of servers to obtain the experiment result. The size of task graph is small, medium and larger, which has the number of task node as 20,100,500 respectively. The number of server types is 5. The computation time is randomly generated from the interval [5, 20], and the communication time is randomly generated from the interval [5, 20]. The out-degree and in-degree of task graph are also randomly generated from the interval [1, 10]. The deadline factor of task graph is set to 0.2, 0.4, 0.6, 0.8, 1.0. Figs 10–12 shows the obtained comparative results for the average NC, as averaged over 50 runs on the same-type servers. According to the contrast analysis of the experimental results in Figs 10 and 11, the average NC of Montage and Epigenomics structure task graph is better than the other structure task graphs by Deadline-DDEP algorithm. For the same size and same deadline of task graph, the CyberShake, LIGO and SIPHT structure task graph have more parallel task nodes; the Deadline-DDEP algorithm will select the CPU faster and price higher of server to schedule the multi-parallel task nodes while meeting their dynamic sub-deadline. From see the contrast analysis of average NC for the large scale task graph in the Fig 12, the average NC of CyberShake, LIGO and SIPHT structure task graph is better than the Montage and Epigenomics structure task graph by Deadline-DDEP algorithm.

The CyberShake, LIGO and SIPHT structure task graph that has the same size and same deadline of task graph, have longer dynamic essential path for entry task node. Every task node will own a tight dynamic sub-deadline by the proposed algorithm. The proposed algorithm will select the CPU faster and price higher of server to schedule every task node while meeting their dynamic deadline, in this way, it makes the total execution cost will be higher.

3) Conclusion. The performance of the proposed algorithm is verified from two aspects. According to the analysis results shown in Figs 5–12, the proposed algorithm exhibits better performance than IC-PCP algorithm, DCCP algorithm and CD-PCP algorithm. Because the proposed algorithm fully considers the total execution cost affected by the dynamic sub-deadline and execution cost of each task node, it makes the sub-deadline and execution cost of all task nodes more reasonable, which can shorten the total execution cost while meeting the user’s defined deadline. The simulation result show that the proposed algorithm has a good performance. According to the analysis results shown in Figs 5–12, the proposed algorithm exhibits better performance than IC-PCP algorithm, DCCP algorithm and CD-PCP algorithm. Because the proposed algorithm fully considers the total execution cost affected by the dynamic sub-deadline and execution cost of each task node, it makes the sub-deadline and execution cost of all task nodes more reasonable, which can shorten the total execution cost while meeting the user’s defined deadline.

## Conclusion

In this paper, we propose a deadline-constrained scheduling algorithm for the cloud computing system based on the driver of dynamic essential path to solve the deadline-constrained task scheduling problem. Because the scheduling model is a DAG model of parallel computing, the algorithm has universality. The innovative points and significance of this paper are as follows. The algorithm adopts the dynamic sub-deadline strategy to solve the problem of the dynamic sub-deadline affected by the change of the dynamic essential path of each task node in the scheduling process. Compared with the existing scheduling algorithm, the dynamic sub-deadline is more reasonable using the proposed strategy, which adds to the planning successful rating. The algorithm uses the quality assessment of optimization cost strategy to solve the selective problem of scheduling server for each task node. The strategy chooses the optimal server that has the lower time and cost quality values by the sub-deadline urgency and the relative execution cost in the scheduling process. The optimal server for each task node can shorten the total execution cost while meeting the user’s defined deadline. The time complexity of the proposed algorithm is *O*(*n*^2^), which is lower than those of the traditional deadline-constrained cloud scheduling algorithms. As a result, the proposed method is simple and viable. Compared with the other deadline-constrained scheduling algorithms, the performance of the proposed algorithm is much better.

In conclusion, the proposed algorithm is able to solve the cloud computing scheduling problem, and offer a certain reference value for solving the scheduling problem of parallel computing, distributed computation and grid computing. Our future work will use multi-objective heuristic algorithm to solve the communication-change application scheduling problem on the Cloud computing and will take into account the load balance.

## Supporting information

S1 File The minimal data set. (RAR)
